# A novel zinc‐binding alcohol dehydrogenase 2 from *Arachis diogoi,* expressed in resistance responses against late leaf spot pathogen, induces cell death when transexpressed in tobacco

**DOI:** 10.1002/2211-5463.12040

**Published:** 2016-02-25

**Authors:** Dilip Kumar, Sakshi Rampuria, Naveen Kumar Singh, Pulugurtha B. Kirti

**Affiliations:** ^1^ Department of Plant SciencesSchool of Life SciencesUniversity of HyderabadIndia

**Keywords:** *Arachis diogoi*, HR‐like cell death, Rossmann fold containing NADB domain, zinc‐binding alcohol dehydrogenase 2

## Abstract

A novel zinc‐binding alcohol dehydrogenase 2 (AdZADH2) was significantly upregulated in a wild peanut, *Arachis diogoi* treated with conidia of late leaf spot (LLS) pathogen, *Phaeoisariopsis personata*. This upregulation was not observed in a comparative analysis of cultivated peanut, which is highly susceptible to LLS. This zinc‐binding alcohol dehydrogenase possessed a Rossmann fold containing NADB domain in addition to the MDR domain present in all previously characterized plant ADH genes/proteins. Transient over‐expression of *AdZADH2* under an estradiol inducible promoter (XVE) resulted in hypersensitive response (HR)‐like cell death in tobacco leaf. However, the same level of cell death was not observed when the domains were transiently expressed individually. Cell death observed in tobacco was associated with overexpression of cell death related proteins, antioxidative enzymes such as SOD, CAT and APX and pathogenesis‐related (PR) proteins. In *A. diogoi, AdZADH2* expression was significantly upregulated in response to the plant signaling hormones salicylic acid, methyl jasmonate, and sodium nitroprusside.

**Abbreviations**
ADH: alcohol dehydrogenase
AdZADH2: *Arachis diogoi*‐zinc binding alcohol dehydrogenase 2
APX: ascorbate peroxidase
CAT: catalase
HIN1: harpin‐induced1 gene
HMG‐CoA R: 3‐hydroxy‐3‐methyl‐glutaryl‐CoA reductase
HSR203J: hypersensitive response related 203j
LLS: late leaf spot
MDR: medium‐length dehydrogenase/reductase
PR: pathogenesis related
RACE: rapid amplification of cDNA ends
SNP: sodium nitroprusside
SOD: superoxide dismutase
XVE: estradiol inducible promoter

Zinc‐binding alcohol dehydrogenases are NAD(P)‐dependent oxidoreductases involved in hydride ion transfer from alcohols to NAD^+^ catalyzing reversible oxidation of alcohols to aldehydes or ketones. They are found in all plants, playing important roles in plant growth, pollen, and seedling development and fruit ripening 1, 2. They are well‐studied members of the medium‐length dehydrogenase/reductase (MDR) protein superfamily 3.

Expression of zinc‐binding alcohol dehydrogenase was induced in Cyanobacterium PCC 6803 upon exposure to different environmental stresses 4 and in citrus upon interaction with *Citrus leprosis* virus 5. Cinnamyl alcohol dehydrogenase (CAD), a zinc‐binding MDR enzyme, is involved in lignin biosynthesis exhibiting defense‐related activity and strengthening of cell walls 6. A zinc‐binding alcohol dehydrogenase is induced in Oomycete plant pathogenic fungus, *Phytophthora infestans* during the infection process 7. Recently, another member of the MDR protein superfamily, an alcohol dehydrogenase gene of the fungus *Metarhizium anisopliae,* was found to be expressed during insect colonization and invasion 8.

In maize, *ADH1* transcripts increase significantly under anaerobic conditions 9. Chung and Ferl 10 demonstrated that transgenic *Arabidopsis* plants expressing ADH promoter–Gus fusions exhibited high Gus expression when grown under conditions simulating anoxia. When these plants are grown in conditions that mimic normal soil growth, the Gus expression was low, showing the relative levels of ADH under anoxia and normal soil conditions. In line with these observations, Ismond *et al*. 11 showed that the *Arabidopsis‐ADH1‐*null mutants exhibited decreased hypoxic survival. Dolferus *et al*. 12, 13 studied the expression of the *ADH* gene in *Arabidopsis* and showed that the gene is induced by dehydration, cold and hypoxia and other environmental stresses. An alcohol dehydrogenase of *Lotus japonicus* (LjADH1) conferred tolerance to H_2_O_2_ induced stress in *E. coli* and yeast cells along with tolerance to heavy metal salts in the latter 14. Myint *et al*. 15 analyzed the expression of alcohol dehydrogenase genes in *Arabidopsis* under polyethylene glycol mediated stress and observed upregulation of *ADH1* and other *ADH* genes in the treatments indicating that the operation of drought tolerance probably involved ethanolic fermentation.

Several wild relatives of peanut including *Arachis diogoi* are highly resistant to many foliar diseases and biotic stresses 16. In an earlier investigation, we have identified a Zinc‐binding alcohol dehydrogenase 2 (*AdZADH2*) gene in *Arachis diogoi* in its interaction with *Phaeoisariopsis personata* that causes the serious late leaf spot disease in the cultivated peanut, *Arachis hypogaea* 17. In this communication, we have focused on the cloning of the novel *AdZADH2* and its involvement in HR‐mediated cell death in tobacco.

## Materials and methods

### 5′/3′ RACE‐PCR, isolation of full length cDNA and transient conditional overexpression of *AdZADH2*

Rapid amplification of cDNA ends (RACE) was performed to clone the full length cDNA of *AdZADH2* by using SMART^™^ RACE cDNA Amplification kit (Clontech, California, USA) using gene specific primers designed from the sequence of a partial fragment identified in an earlier study using cDNA‐AFLP 17. The ORF was amplified with primers *AdZADH2*‐ApaI‐F and *AdZADH2*‐SpeI‐R containing *Apa*I and *Spe*I restriction enzymes, respectively, by using Phusion^™^ DNA polymerase (Finnzymes, Loughborough, UK, NEB, Table S1), cloned in pTZ57R/T and sequenced. The open reading frame (ORF) of the *AdZADH2* with flanking *Apa*I and *Spe*I sites was digested with appropriate restriction enzymes and cloned into pER8 vector (Gifted by N.‐H. Chua, Rockefeller University, USA) to obtain the recombinant pER8:*AdZADH2*. We further cloned both the domains separately in pER8 vector as NADB Rossmann domain D1 (pER8:AdZADH2‐D1) and MDR domain D2 (pER8:AdZADH2‐D2) by using specific primers (AdZADH2‐ApaI‐F and AdZADH2‐D1‐SpeI‐R for D1 and AdZADH2‐D2‐ApaI‐F and AdZADH2‐SpeI‐R for D2) to obtain recombinant vectors pER8:AdZADH2‐D1 and pER8:AdZADH2‐D2 respectively (Fig. S5). Primer sequences used in this study are listed in Table S1.

### Agro‐infiltration, chemical treatment, and cell death assessment

The vectors were mobilized into *Agrobacterium* strain LBA4404 using freeze thaw method. *Agrobacterium* strains harboring different binary vector constructs were grown in Luria Broth in the presence of respective antibiotics, pelleted, resuspended and infiltrated as described earlier 18, 19, 20. After the observation of cell death phenotype, the samples were collected, quick‐frozen in liquid nitrogen and stored at −80 °C for further analysis.

### Cell death quantification and ion leakage experiment

The extent of cell death was quantified by Evans Blue staining following Baker and Mock 21 method. For electrolyte leakage analysis, leaf discs (8 mm diameter) were cut off from agro‐infiltrated regions after 48 h post infiltration using a cork borer and transferred to distilled water containing 30 μm 17β‐estradiol in Petri dishes under bright light with 14/10 h photoperiod 17, 22, 23. Electrolyte leakage values were determined by using an electro‐conductivity meter (Digisun Electronics, Hyderabad, India) at different time intervals and data were plotted from three biological replicates.

### Quantitative RT‐PCR analysis post estradiol treatment

Leaves were infiltrated with agrobacterium strain LBA4404 harboring pER8, pER8:AdZADH2, pER8:AdZADH2‐D1, and pER8:AdZADH2‐D2 vectors, respectively. Total RNA was isolated from leaf samples with and without estradiol treatment collected at 0, 24, and 48 h time points. First strand cDNA was synthesized and used in the analysis of the transcript levels for several defense‐associated genes along with HR marker genes using gene specific primers (Table S2). Actin was used as internal control for calculating relative gene expression. Relative fold change in RNA expression was estimated using ΔΔ*C*_T_ method 24.

### Hormonal and SNP treatments

Twigs from the field‐grown plants were cut and kept in a tray for 2 weeks on moist cotton saturated with sterile distilled water at the base and the tray was covered with a polythene sheet to maintain humidity for adventitious root formation and recovery. For various chemical treatments, the rooted twigs were kept in the corresponding solution. The treatments given were 100 μm salicylic acid (SA), 100 μm methyl jasmonate (MeJA), 100 μm abscisic acid (ABA), 250 μm ethephon, 100 μm sodium nitroprusside (SNP), a combination of 100 μm each of salicylic acid and methyl jasmonate. A mock treatment with water served as control. The treatments were carried out at different time intervals up to 24 h and incubated in a growth room at 27 ± 1 °C under 14/10 h photoperiod provided by light intensity of 30 μmol·m^−2^·s^−1^. Samples were collected at regular intervals, quickly frozen in liquid nitrogen, and stored at −80 °C until use.

### Expression analysis of *AdZADH2* using qRT‐PCR

Total RNA, isolated from control‐ and hormone‐treated peanut samples was treated with RNase free DNase1 (Sigma‐Aldrich, Carlsbad, CA, USA) to eliminate any DNA contamination and was reverse transcribed with oligo‐dT primer using SMART^™^ MMLV Reverse Transcriptase (Clontech, Becton Dickinson, Palo Alto, CA, USA). cDNA samples were diluted fivefold and 0.5 μL of the diluted reaction mixture was taken as qRT‐PCR template in a 20 μL total reaction volume containing 0.4 μm gene specific primers with10 μL SYBR Premix Ex Taq with ROX (Takara Bio Inc., Kusatsu, Shiga, Japan) and the samples were appraised in three technical replicates. PCR analysis was carried out in Realplex amplifier (Eppendorf, Hamburg, Germany) with the following cycle parameters: 95 °C for 5 min; 40 cycle of 95 °C for 20 s, 58 °C for 20 s, 72 °C for 20 s followed by melting curve. Gene specific primers were designed from 3′ UTR of *AdZADH2* (Table S1). Polyubiquitin (UBI1) and alcohol dehydrogenase III (*ADH3*) were used as internal controls for calculating relative quantification of gene expression 25, 26. Relative fold change in RNA expression was estimated using ΔΔ*C*_T_ method 24.

## Results and discussion

### *AdZADH2* sequence analysis

**Figure 1:**
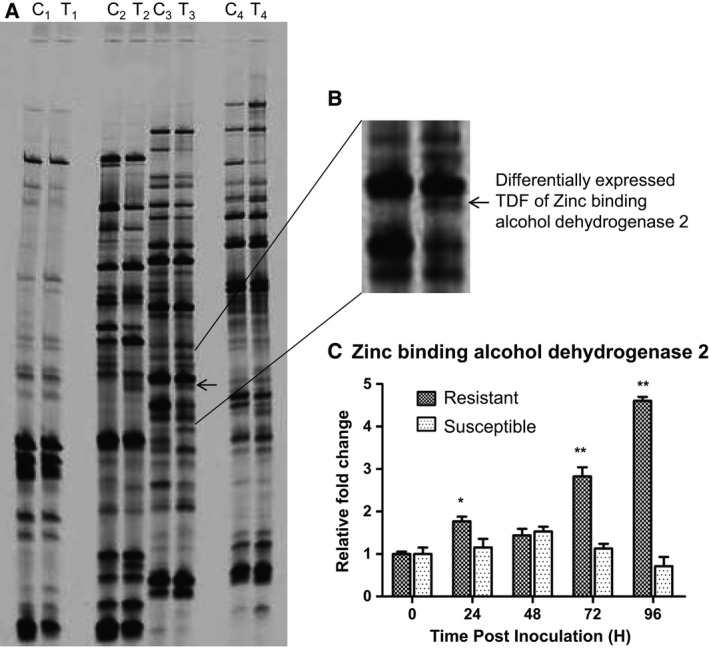
(A) cDNA‐AFLP gel picture showing differential expression of TDFs in *Arachis diogoi* upon pathogen challenge. C‐ represent the pool sample of mock inoculated at 0, 24, 48, 72, and 96 h while T‐ represent the pool sample of pathogen inoculated at 0, 24, 48, 72, and 96 h. The primer combinations used were; Lane C_1_T_1_: M‐CTC/E‐AGG, C_2_T_2_: M‐CTC/E‐ACG, C_3_T_3_: M‐CTC/E‐AGC, C_4_T_4_: M‐CTC/E‐ACC. Arrowhead represents differentially expressed TDF of *AdZADH2*. (B) Enlarged view of differentially expressed transcripts derived fragment of zinc‐binding alcohol dehydrogenase 2. (C) Quantitative PCR analyses of *AdZADH2* in response to *Phaeoisariopsis personata* inoculation. Leaf tissues for both pathogen and mock inoculated plants at 0, 24, 48, 72, and 96 hpi were used and relative gene expression was calculated by comparative ΔΔ*C*_T_ method. Data were normalized to the Polyubiquitin (UBI1) and Alcohol dehydrogenase‐3 (*ADH‐3*) expression level as internal reference genes and data were plotted from three independent experiments mean ± SD. Statistical analysis was performed with Student's *t*‐test, asterisks represents a significant difference from the susceptible counterpart (*P < 0.05, **P < 0.01). *Arachis diogoi* (Resistant) and *Arachis hypogaea* cv.JL‐24 (Susceptible).

A partial fragment of a zinc‐binding alcohol dehydrogenase 2 was differentially upregulated in a cDNA‐AFLP analysis of an interaction between *Arachis diogoi* and the late leaf spot pathogen *P. personata* 17. It was made full length using 3′/5′ RACE. The cDNA of *AdZADH2* comprised an ORF of 1902 bp (Fig. S1) and codes for a polypeptide of 633 amino acids (GenBank Accession number KT321126). Its upregulation under fungal treatment has been revalidated in a comparative real time PCR analysis of the resistant wild peanut and its susceptible cultivated peanut counterpart (Fig. 1C); its upregulation under pathogen challenge was observed only in the wild peanut.

Multiple sequence alignment and phylogenetic analyses displayed its close relation in plants to soybean predicted zinc‐binding ADH2‐like protein (different from ADH2 studied 27, 28, 29) with 85% sequence similarity (Fig. S2a). AdZADH2 shows blastp specific hit of Mgc45594_like in MDR domain region (Table S3) with an undetermined function. Psort (http://psort.hgc.jp/form.html) identifies AdZADH2 as a resident of peroxisomes (probability of 85.2%); moreover, PredPlantPTS1 (http://ppp.gobics.de) predicted the presence of a C‐terminal PTS1 domain (tripeptide AKL) for peroxisome targeting (probability 100.0%). This C‐terminal signal for peroxisome translocation is absent in previously studied ADH proteins. Moreover, we made multiple sequence alignment between AdZADH2 with some previously studied ADH proteins using network protein sequence analysis (NPS, https://npsa-prabi.ibcp.fr/cgi-bin/align_multalin.pl, 30. Interestingly, the NADB Rossmann domain was absent in other plant ADH proteins studied so far (Fig. S4) showing that it is a novel ADH2. The predicted molecular weight and p*I* of AdZADH2 protein were found to be totally different from ADH proteins studied earlier (Table S3).

A protein blast analysis of AdZADH2 revealed two conserved domains, a NADB Rossmann domain and MDR domain (Fig. S2A,B). These domains are reportedly involved in various activities such as oxidoreductases, catalysis, and enzymatic reactions providing catalytic and structural stability to the protein 3, 27. Rossmann fold in NAD‐dependent dehydrogenases found in many protein superfamilies (mostly oxidoreductases). It decides the specificity of hydride transfer thereby providing NAD or NADP as a cofactor to MDR domain for interconversion of ethanol and acetaldehyde. Interestingly NADB Rossmann domain of AdZADH2 was not much conserved as it showed just 83%, 72%, and 64% homology with NADB domains soybean (*Glycine max*; XP_003518845.1), tobacco (*Nicotiana tomentosiformis*; XP_009612756.1) and *Arabidopsis* (*Arabidopsis thaliana*; NP_175390.2), respectively; however, MDR domain was found to be highly conserved (Fig. S2).

Plant alcohol dehydrogenases (ADH) have been well characterized for their role in cell survival under hypoxic and anaerobic conditions. During anaerobic glycolysis i.e., fermentation, ADH diverts the formation of lactic acid from pyruvate to less toxic and more diffusible ethanol, thereby helping cells to survive in the absence of oxygen required for normal respiration. Available evidences suggest that plant ADH gene family comprises survivors of multiple rounds of gene expansion and contraction resulting in gains in roles like production of characteristic scents that act to attract animals, pollinators, or agents of seed dispersal and to protect plants against herbivores besides anaerobic fermentation 1, 2.

### Conditional expression of *AdZADH2* in tobacco results in HR‐like cell death

**Figure 2:**
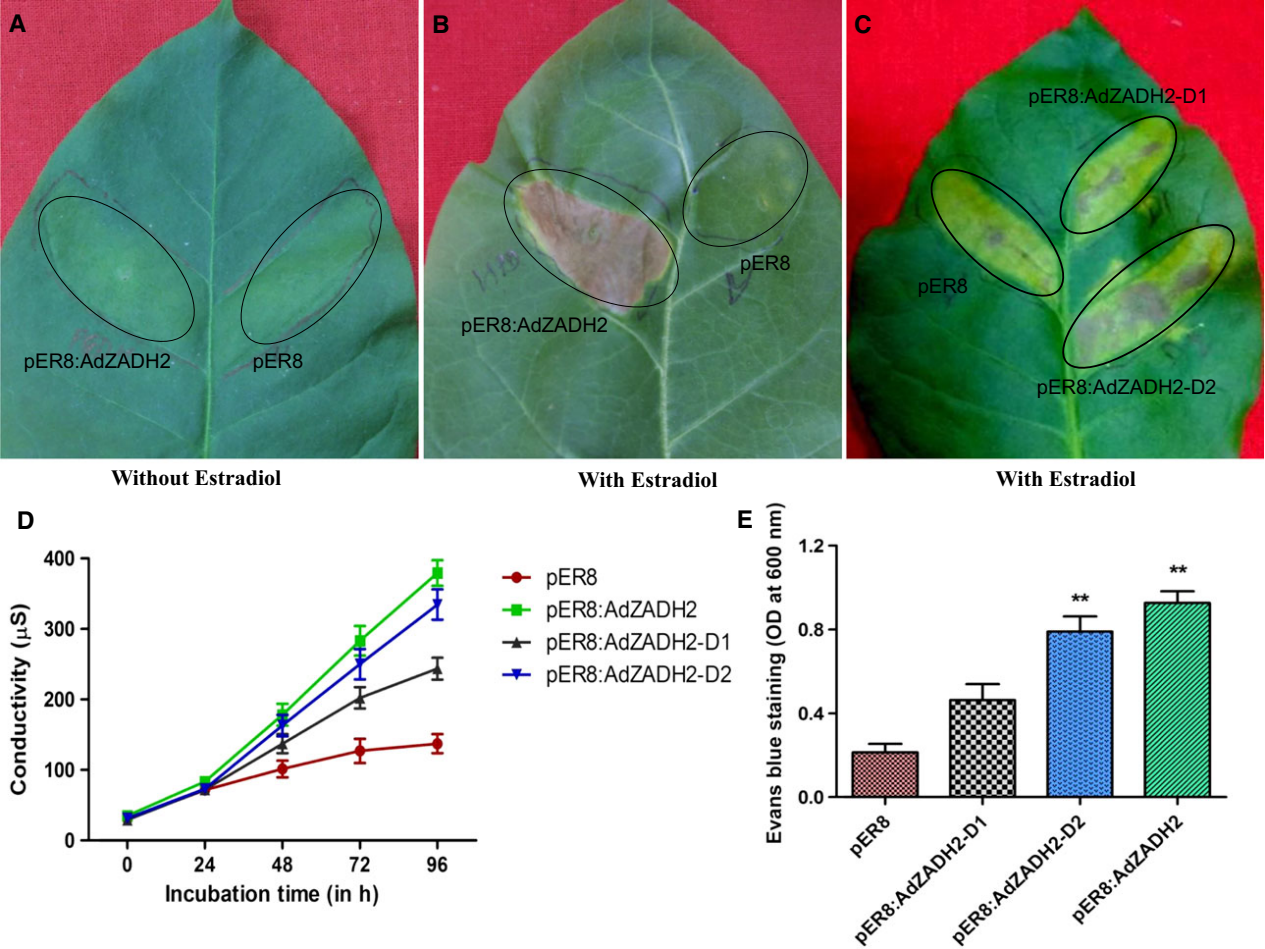
Zinc‐bindidng alcohol dehydrogenase 2 induced cell death in tobacco leaf upon conditional expression. Tobacco leaf areas were infiltrated with the *Agrobacterium* strain, LBA4404 carrying the empty vector pER8, pER8:*AdZADH2*, pER8:*AdZADH2*‐D1 and pER8:*AdADH2*‐D2. Transgene expression was induced by the application of 30 μm 17β‐estradiol after 48 hpi to one (B) and no induction has been given to another (A) and photographs were taken after 72–96 hpi. (C) Domain specific induction of HR‐like cell death of NADB Rossmann (D1) and MDR domain (D2) in comparison to control (pER8) and pER8:*AdZADH2*. (D) Represents ion leakage analysis of *AdZADH2* induced cell death in response to 30 μm estradiol treatment in comparison to control (pER8), pER8:*AdZADH2*‐D1 and pER8:*AdADH2*‐D2. Five leaf discs (8 mm diameter) were collected from agro‐infiltrated areas of corresponding regions and incubated in 5 mL of water containing 30 μm 17β‐estradiol and ion leakage was measured upto 96 h by digital conductivity meter. Four biological replicates mean ± SD data were plotted. (E) *AdZADH2* induced cell death in comparison to control (pER8), pER8:*AdZADH2*‐D1 (NADB Rossmann domain) and pER8:*AdADH2*‐D2 (MDR domain) was quantified using Evans blue staining. Evans blue uptake after 72 h post estradiol was quantified using spectrophotometry. Three biological experiments mean ± SD data were plotted. (**P < 0.01).

**Figure 3:**
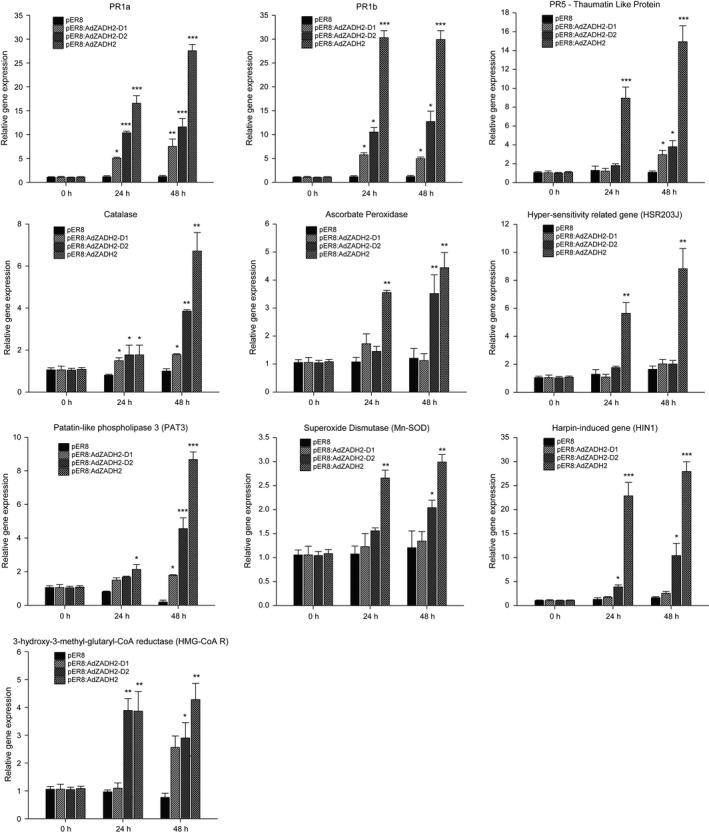
Relative expression of defense‐associated genes and cell death related genes in leaves transiently expressing pER8:*AdZADH2,* pER8:*AdZADH2*‐D1 and pER8:*AdADH2*‐D2 in relation to control (pER8) after estradiol application at 0, 24, and 48 h. Quantitative RT‐PCR analysis was performed using total RNA from samples collected at various time points. Relative expression was calculated by comparative ΔΔ*C*_T_ method. Statistical analysis was performed with student's *t*‐test, asterisks indicate upregulated expression relative to control samples (*P < 0.05, **P < 0.001, ***P < 0.0001). Gene specific primers were used for amplification of different genes with Actin serving as internal reference gene. The primers sequence used in the study were given in Table S2.

Transient expression of *AdZADH2* in detached leaves of tobacco under an estradiol inducible promoter (XVE) 31, 32 resulted in hypersensitive response (HR) like cell death in the infiltrated area 48–72 h postestradiol treatment (Fig. 2A,B). Induced expression of *AdZADH2* was observed to be strong at 24 and 48 h postinfection (hpi). This strong expression of *AdZADH2* was found to be associated with enhanced expression of defense‐related genes (*NtPR1a, NtPR1b*, and *NtPR5‐TLP*). Along with the expression of genes for pathogenesis‐related proteins, marker genes for cell death such as *NtPAT3*,* HMG‐CoA R*,* HSR203J*, and *HIN1* and antioxidant enzymes such as catalase (CAT), ascorbate peroxidase (APX), and superoxide dismutase (Mn‐SOD) also got upregulated in the infiltrated regions analyzed by quantitative RT‐PCR up to 48 h postestradiol application (Fig. 3). *HMG‐CoA R*,* HSR203J*, and *HIN1* were found to be co‐upregulated at 24 and 48 h of treatment. *HIN1* and *HSR203J* have been shown to be closely associated with cell death in incompatible interactions in tobacco and were used as markers for HR 32, 33, 34. Along with HR marker genes, we have also observed the upregulation of *PAT3* gene; transient expression of a patatin like phospholipase in tobacco resulted in the accumulation of fluorescent phenolics, pathogenesis‐related proteins CaPR1, CaSAR82A, and ROS leading to cell death in pepper leaves 35. Expression profiling postfungal treatment in *Populus* *trichocarpa* showed possible involvement of *CAD/CAD‐*like genes in plant development and defense against various pests and pathogens 36. The accelerated cell death gene, *acd11 of Arabidopsis* constitutively expresses the defense‐related genes that are associated with hypersensitive response normally triggered by avirulent pathogens 37.

There is evidence of the involvement of ADH in plant fungal interactions through the activation of fermentative pathway. Pathogen infection, particularly biotrophic pathogens, leads to a condition of reduced oxygen pressure that leads to a fermentation pathway and the development of reactive oxygen species. Under such conditions, upregulation of ADH has been reported in biotrophic pathogen infections. Proels *et al.,* 38 have observed that the barley seedlings treated with the biotrophic pathogen, *Blumeria graminis* f.sp *hordei* upregulated ADH1 and ADH2, but not ADH3. It has been surmised that the pathogen infection might reduce photosynthetic activity in the infected tissues leading to reduced oxygen pressure resulting in a condition similar to anoxia and inducing the fermentation pathway to compensate the reduced energy levels. Moreover, ethanolic fermentation (a major means of ATP production under anaerobic condition in plants) leads to the accumulation of high levels of acetaldehyde, which is a strong cell toxin. It binds to nucleic acids and proteins forming stable acetaldehyde‐protein adducts, eventually leading the cell toward PCD 39, 40. Uehara *et al.,* 41 analyzed the differentially upregulated *ADH* gene in resistant and susceptible cultivars of tomato in response to the challenge from the cyst nematode and observed that there was differential upregulation of the *ADH* transcripts; incompatible interaction showed upregulation and compatible interaction evidencing downregulation of *ADH*. Hren *et al.,* 42 observed the induced expression of sucrose synthase and alcohol dehydrogenase in resistant varieties of grapevine infected by phytoplasma with the conversion of sucrose to alcohol through alcohol fermentation.

Cell death phenomenon was associated with the enhanced expression of defense‐related genes, cell death marker genes and antioxidant enzymes (Fig. 3). Cell death was quantified by using Evans Blue and an ion leakage experiment 43. Cell death with enhanced electrolyte leakage was significantly high in pER8:AdZADH2 regions in comparison to the empty vector infiltrated areas (Fig. 2D,E). The AdZADH2 protein carries two domains, a NADB Rossmann superfamily domain and a MDR Superfamily domain. Furthermore, to see if the cell death could be specifically associated with one of the two domains, we have cloned these domains [as both of them carry separate start (Met) codons] separately under the estradiol inducible promoter in pER8 as NADB Rossmann domain D1(pER8:*AdZADH2*‐D1) and MDR domain D2 (pER8:*AdZADH2*‐D2). We found that after chemical induction, infiltrated area expressing MDR domain (pER8:*AdZADH2*‐D2) showed significantly enhanced cell death in comparison to NADB Rossmann domain (pER8:*AdZADH2*‐D1) (Fig. 2C). In addition, electrolyte leakage analysis and cell death quantified by Evans Blue also supported this observation, where enhanced ion leakage and cell death have been observed with MDR domain in comparison to NADB Rossmann domain (Fig. 2D,E). However, the level of cell death induced by individual domains was less when compared with the complete protein, AdZADH2. Taken together, these results imply that transient expression of AdZADH2 induces cell death and MDR domain played a major role in this phenomenon probably in an interaction with the NADB domain (the expression of which also induced cell death, though not that extent as the MDR domain). Rossmann fold being a NAD‐dependent dehydrogenase dictates the specificity of hydride transfer thereby providing NAD or NADP as a cofactor to MDR domain for interconversion of ethanol and acetaldehyde. Therefore, in the absence of Rossmann fold, MDR domain is probably not able to produce the same level of HR‐like cell death as produced when both domains are present as in *AdZADH2* owing to the reduced availability of NAD or NADP as a cofactor. Quantitative expression analysis shows overexpression of defense related genes, HR marker genes and ROS generated enzymes upon transient overexpression of *pER8:AdZADH2* (Fig. 3); however, the relative fold changes were significantly less when these two domains were overexpressed individually. Moreover, changes in the expression level of HR marker genes viz. *HIN1*,* HSR203J*, and *HMG‐CoA R* were not significant when Rossmann fold was overexpressed alone.

**Figure 4:**
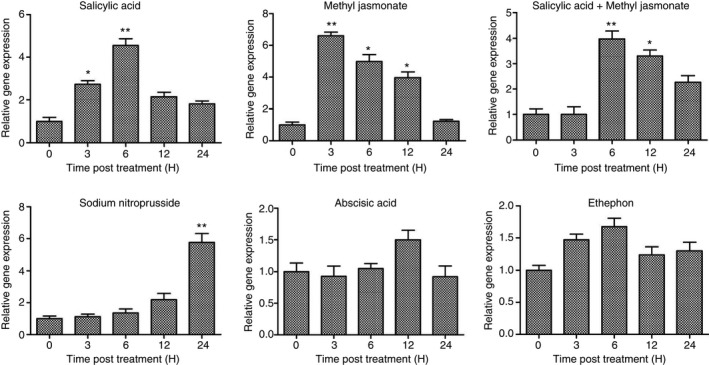
RNA expression of zinc‐binding alcohol dehydrogenase 2 in response to stress hormones. A quantitative RT‐PCR was performed using total RNA isolated from samples collected at different time points (in hours) involving treatments with Salicylic acid (SA), Methyl jasmonate (MeJA), Salicylic acid and Methyl jasmonate together, Abscisic acid (ABA), Ethephon and Sodium nitroprusside (SNP). Relative transcript expression was calculated by comparative ΔΔ*C*_T_ method. For qPCR analysis primers were designed from 3′ UTR of *AdZADH2*. Data were normalized to the Polyubiquitin (UBI1) and Alcohol dehydrogenase‐3 (*ADH‐3*) level as internal reference genes and plotted from three independent experiments. Statistical analysis was performed with student's *t*‐test, asterisks indicates significant up‐accumulation in relative to mock sample (*P < 0.05, **P < 0.001).

### Expression analysis of *AdZADH2* in response to various signaling molecules

Salicylic acid and methyl jasmonate are the important signaling molecules in systemic acquired resistance (SAR) and wound signaling, respectively, and the expression of *AdZADH2* showed strong accumulation in transcripts in salicylic acid and methyl jasmonate‐treated samples at early time points. *AdZADH2* appeared to be regulated primarily by salicylic acid as the induced expression at 3 h stage in the combination treatment of SA and MeJA exhibited down regulation of the transcripts in comparison to 3 h MeJA treatment. Nitric oxide (NO) is an emerging essential component of plant defense signaling and SNP treatment was used at different time intervals. We observed a significant increase in *AdZADH2* transcript levels at 12 and 24 h of SNP treatment. No significant expression of the gene was observed in treatments with ABA, the major signaling molecule for abiotic stress responses and ethephon. ADH genes were reportedly expressed by abscisic acid 44. Echave *et al*., 45 studied an alcohol dehydrogenase in detail and proposed that ADHE having aldehyde dehydrogenase (ALDH) and dehydroquinate (DHQ) _Fe_ADH domain scavenges H_2_O_2_ in *E. coli* cells grown under aerobic conditions. In case of ethephon treatment, we found a trivial expression pattern of *AdZADH2* at all‐time points indicating that its expression is also not controlled by ethephon (Fig. 4). Thus, plants try to defend themselves from the pathogen attack and herbivory by activating a number of signaling molecules such as JA, SA and SNP, which share some common signaling components 46. Though, SA has been initially thought to attenuate the JA pathway, subsequent analyses have conclusively proved the coordination between both the signaling molecules, which are interconnected in the plant to have economization of energy to combat various stresses that attack it 47.

Nitric oxide controls a signaling cascade that regulates plant responses to developmental processes, biotic and abiotic stress 48, 49, 50, 51. We have found induced expression in the *AdZADH2* transcripts at late stage of SNP treatment. An interaction between hydrogen peroxide and nitric oxide is shown to be associated with several forms of cell death 52, 53. *AdZADH2* appears to support nitric oxide (NO) mediated HR cell death; NO production leads to the production of H_2_O_2_ (instead of ROS), which in turn results in HR‐mediated PCD 54. Several lines of evidence have shown that the early nitric oxide (NO) burst in host plant cells after pathogen attack functions as a major defense response associated with resistance in plant pathogen interaction 55. NO is an interesting molecule that is used by microorganisms in pathogenesis and host cells to activate the immune system to counter the pathogen challenge 56, 57. Thus, the significant upregulation of *AdZADH2* by the resistant host against the LLS pathogen attack under SNP treatment could be justified as a resistance response.

## Conclusion

Peroxisomal resident novel *AdZADH2* protein was upregulated in *A. diogoi* in treatment with *P. personata*. Conditional overexpression of *AdZADH2* in tobacco leaf under an estradiol inducible promoter (XVE) resulted in HR‐like cell death associated with an enhanced electrolyte leakage and enhanced expression of defense‐related genes (*NtPAT3*,* NtPR1a*,* NtPR1b*,* and NtPR5*), marker genes for cell death such as *HMGR*,* HSR203J*, and *HIN1* and antioxidant enzymes such as CAT, APX, and SOD. The expression of *AdZADH2* was upregulated post‐SA, JA, and SNP treatments in *A. diogoi*. This report shows involvement of Zn containing ADH protein having NADB Rossmann fold and MDR domain in HR‐like cell death in plants. Cell death activities are not domain additive, but are probably a result of interaction between these two domains; cell death was significantly decreased when the two domains are individually overexpressed.

## Author contributions

PBK and DK conceived and designed the project, SR, DK, and NKS acquired the data, where NKS identified full gene sequence of AdZADH2 by RACE PCR. SR and DK performed experiments related to HR and real‐time expression studies. PBK, SR and DK analyzed, interpreted the data, and wrote the paper.

## Supporting information


**Table S1.** Oligonucleotides sequence used in the study.
**Table S2.** Primers and their sequences used in study for semi‐quantitative and quantitative PCR Analysis.
**Table S3.** A list of previously studied ADH genes.
**Figure S1.** Cloning of AdZADH2 from *Arachis diogoi*.
**Figure S2.** Multiple sequence alignment of the deduced amino acid sequence of AdZADH2 with closely related uncharacterized Zinc‐binding alcohol dehydrogenase sequences.
**Figure S3.** Phylogenetic relationship of AdZADH2 with other zinc‐binding alcohol dehydrogenase family members.
**Figure S4.** Multiple sequence alignment of the deduced amino acid sequence of AdZADH2 with some previously studied zinc‐binding alcohol dehydrogenase sequences from other organisms was done using default parameters of network protein sequence analysis (NPS). NADB Rossmann fold is absent in previously studied ADH proteins; moreover MDR domain of AdZADH2 shows no significant homology with other ADH proteins in the genes used in the alignment were from *Zea mays* (ZmADH1, ZmADH2), *Oryza sativa* (OsADH1), *Solanum lycoperisicum* (SlADH1), *Arabidopsis thaliana* (AtADH1, AtADH2), *Glycine max* (GmADH1, GmADH2), and *Lotus japonicus* (LjADH1), respectively. Accession numbers are given in Table S3.
**Figure S5.** Cloning of NADB Rossmann domain and MDR superfamily domain.
**Figure S6.** Transient constitutive expression of *AdZADH2* induced cell death in tobacco leaf upon constitutive expression under 35S promoter.
