# Supplementary material for: A novel zinc‐binding alcohol dehydrogenase 2 from Arachis diogoi, expressed in resistance responses against late leaf spot pathogen, induces cell death when transexpressed in tobacco
Source: FEBS Open Bio. 2016 Feb 25;6(3):200–10. doi: 10.1002/2211-5463.12040 (PMC4794784; doi:10.1002/2211-5463.12040)
Supplement: Supplementary file 1 — Table S1. Oligonucleotides sequence used in the study. Table S2. Primers and their sequences used in study for semi‐quantitative and quantitative PCR Analysis. Table S3. A list of previously studied ADH genes. Figure S1. Cloning of AdZADH2 from Arachis diogoi. Figure S2. Multiple sequence alignment of the deduced amino acid sequence of AdZADH2 with closely related uncharacterized Zinc‐binding alcohol dehydrogenase sequences. Figure S3. Phylogenetic relationship of AdZADH2 with other zinc‐binding alcohol dehydrogenase family members. Figure S4. Multiple sequence alignment of the deduced amino acid sequence of AdZADH2 with some previously studied zinc‐binding alcohol dehydrogenase sequences from other organisms was done using default parameters of network protein sequence analysis (NPS). NADB Rossmann fold is absent in previously studied ADH proteins; moreover MDR domain of AdZADH2 shows no significant homology with other ADH proteins in the genes used in the alignment were from Zea mays (ZmADH1, ZmADH2), Oryza sativa (OsADH1), Solanum lycoperisicum (SlADH1), Arabidopsis thaliana (AtADH1, AtADH2), Glycine max (GmADH1, GmADH2), and Lotus japonicus (LjADH1), respectively. Accession numbers are given in Table S3. Figure S5. Cloning of NADB Rossmann domain and MDR superfamily domain. Figure S6. Transient constitutive expression of AdZADH2 induced cell death in tobacco leaf upon constitutive expression under 35S promoter. [file FEB4-6-200-s001.docx]

**Supplementary Materials**

**Table S1** List of oligonucleotides sequence used in the study

| **Oligo Name** | **Oligo Sequence (5'- 3')** |
| --- | --- |
| 5'AdZADH2-GSP1  3'AdZADH2-GSP1  AdZADH2-3'UTR-F  AdZADH2-3'UTR-R  AdZADH2-ApaI-F  AdZADH2-SpeI-R  AdZADH2-D1-SpeI-R  AdZADH2-D2-ApaI-F | TCCTCACTATTCTGGTAGCTTTCC  CTGTCAGTGACTTGAAGGTTGG  CTCCACTCAGGCAAAAGTGTTGG  GCTGCAAACTCGAGCTCTCC  AGGGCCCATGGAGATCAAACCTGG  CACTAGTTCATAATTTGGCCACTTGCTTC  ATACTAGTTCAACCTCGACGATTTG  TAGGGCCCATGGAGTACTGGCCA |

**Table S2** Primers names and their sequences used in study of AdZADH2 for semi-quantitative and quantitative PCR analysis

| **Gene Name** | **Primer Abbreviation** | **Primer Sequence (5'- 3')** |
| --- | --- | --- |
| *NtPR1a* | NtPR1a-F  NtPR1a-R | ATGGTCAATACGGCGAAAAC TAGCACATCCAACACGAACC |
| *NtPR1b* | NtPR1b-F  NtPR1b-R | ACAACGGGTAGCAGCCTAT  TAGCACATCCAACACGAACC |
| *NtPR5* Thaumatin like protein | NtPR5TLP-F  NTPR5TLP-R | ACCCAATCAGGACTTTGTCG AGCATCAGGGCATCTTTCC |
| *NtMn-SOD* | SOD Mn-F  SOD Mn-R | TCCCCTACGACTATGGAGCA  CGGTATGCAATTTGGCGACG |
| *NtAPX* | NtAPX-F  NtAPX-R | GTTTGGGCTTTTCTCCTCGAC  GGAGCATAAGAGGAGCGCAA |
| *NtCAT* | NtCAT-F  NtCAT-R | GGCCGCTACAACTCTCTCTTT  ACAGGACCTCTTGCACCAAC |
| *NtPAT3* | NtPAT3-F  NtPAT3-R | ATTTGACTGGAGAGGCTGCG TGACCCTTGACACTGGCTTC |
| *NtHMGR* | NtHMGR-F  NtHMGR-R | TCTTCGTTGAGGATCCCTTG AGTTCCCAGATATGCCGATG |
| *NtHIN1* | NtHIN1-F  NtHIN1-R | CAGCTTGCGTCCAGTATTCA GGCATCTGGTTTCCTCAAAA |
| *NtHSR203J* | NtHSR203J-F  NtHSR203J-R | TATCCGGCTGGCTTACAGTT ACGGAATTGTCGTTTCGTTC |
| *Actin* | Actin-F  Actin-R | TGGCATCACACTTTCTACAA  CAACGGAATCTCTCAGCTCC |
| *UBI1* | UBI1RT-F  UBI1RT-R | TCTTGTCCTCCGTCTTAGGG  AGCAAGGGTCCTTCCATCTT |
| *ADH-3* | AdRTADH3-F  AdRTADH3-R | GACGCTTGGCGAGATCAACA  AACCGGACAACCACCACATG |

**Table S3** List of previously studied ADH genes

| **Name** | ***Source*** | **Uniprot ID /Gene ID** | **Length of protein (aa)** | **Mw of protein (kDa)** | **pI** | **Fe/Zn** | **Blastp specific hit for MDR domain** | **Publications** |
| --- | --- | --- | --- | --- | --- | --- | --- | --- |
| ZADH2 | *Arachisdigoi* | KT321126 | 634 | 68.64 | 9.17 | Zn | Mgc45594_like | This study |
| ADH1 | *Arabidopsis thaliana* | PO6525 | 379 | 41.17 | 5.83 | Zn | Alcohol_DH_Plants | Noguchi et al. 2001, Ismond et al. 2003 [1,2] |
| ADH2 | *Arabidopsis thaliana* | Q96533 | 379 | 40.69 | 6.51 | Zn | Alcohol_DhH_Class III | Martinez et al. 1996 [3] |
| ADH1 | *Vitisvinifera* | Q43690 | 380 | 41.05 | 6.2 | Zn | Alcohol_DH_Plants | Hren et al. 2009 [4] |
| ADH2 | *Vitisvinifera* | Q9FZ01 | 380 | 41.19 | 5.85 | Zn | Alcohol_DH_Plants | Tesniere and Verries 2000 [5] |
| ADH3 | *Vitisvinifera* | Q9FZ00 | 382 | 41.24 | 6.76 | Zn | Alcohol_DH_Plants | Tesniere and Verries 2000 [5] |
| ADH1 | *Zea mays* | P00333 | 379 | 40.98 | 6.28 | Zn | Alcohol_DH_Plants | Gerlach et al. 1982, Osterman and Dennis 1989 [6,7] |
| ADH2 | *Zea mays* | P04707 | 379 | 41.05 | 5.71 | Zn | Alcohol_DH_Plants | Dennis et al. 1985 [8] |
| ADH1 | *Glycine max* | O82478 | 374 | 40.24 | 6.08 | Zn | Alcohol_DH_Plants | Newman and Vantoai 1992, Paul and Ferl 1998 [28, 29] |
| ADH2 | *Glycine max* | Q9ZT38 | 341 | 36.38 | 6.13 | Zn | Alcohol_DH_Plants | Newman and Vantoai 1992, Paul and Ferl 1998 [9,10] |
| ADH1 | *Lotus japonicus* | G8JZ50 | 380 | 41.09 | 5.92 | Zn | Alcohol_DH_Plants | Zeng et al. 2011 [11] |
| ADH2 | *Solanumlycopersicum* | P28032/ M86724 | 380 | 41.04 | 6.02 | Zn | Alcohol_DH_Plants | Uehara et al. 2010 [12] |
| ADH2 | *Oryza sativa* | Q0ITW7 | 379 | 41.21 | 6.03 | Zn | Alcohol_DH_Plants | Xie and Wu 1989 [62] |
| ADH1 | *Oryza sativa* | B6F2C1 | 379 | 40.98 | 6.2 | Zn | Alcohol_DH_Plants | Matsumura et al. 1998, Xie and Wu 1989, Takahashi et al. 2011 [13-15] |
| ADH1 | *Hordeumvulgare* | P05336 | 379 | 40.88 | 6.15 | Zn | Alcohol_DH_Plants | Good et al. 1988 [16] |
| ADH2 | *Hordeumvulgare* | P10847 | 373 | 40.51 | 5.5 | Zn | Alcohol_DH_Plants | Trick et al. 1988 [17] |
| ADH3 | *Hordeumvulgare* | P10848 | 379 | 41.01 | 6.08 | zn | Alcohol_DH_Plants | Hanson et al. 1984 [18] |
| ADH1 | *Pennisetumglaucum* | P14219 | 379 | 40.91 | 6.28 | Zn | Alcohol_DH_Plants | Gaut and Clegg 1991 [19] |
| ADH1 | *Pisumsativum* | P12886 | 380 | 41.15 | 6.08 | Zn | Alcohol_DH_Plants | Llewellyn et al. 1987 [20] |
| ADH1CN | *Phaseolusacutifolius* | Q43015 | 380 | 40.91 | 5.87 | Zn | Alcohol_DH_Plants | Garvin et al. 1994 [21] |
| ADH srl1192 | *Synechocystis sp.* (strain PCC 6803 / Kazusa) | P74721 | 336 | 35.86 | 5.68 | Zn | CAD1 (cinnamyl alcohol dehydrogenase 1) | vidal et al. 2009 [22] |
| ADHE | *Escherichia Coli* | P0A9Q7 | 891 | 96.12 | 6.32 | Fe | ALDH_SF and DHQ_Fe_ADH | Echave et al. 2003 [23] |

**Figure S1** Cloning of AdZADH2 from *Arachisdiogoi*.Representative gel picture of 5' and 3' RACE PCR product of *AdZADH2* and its open reading frame.


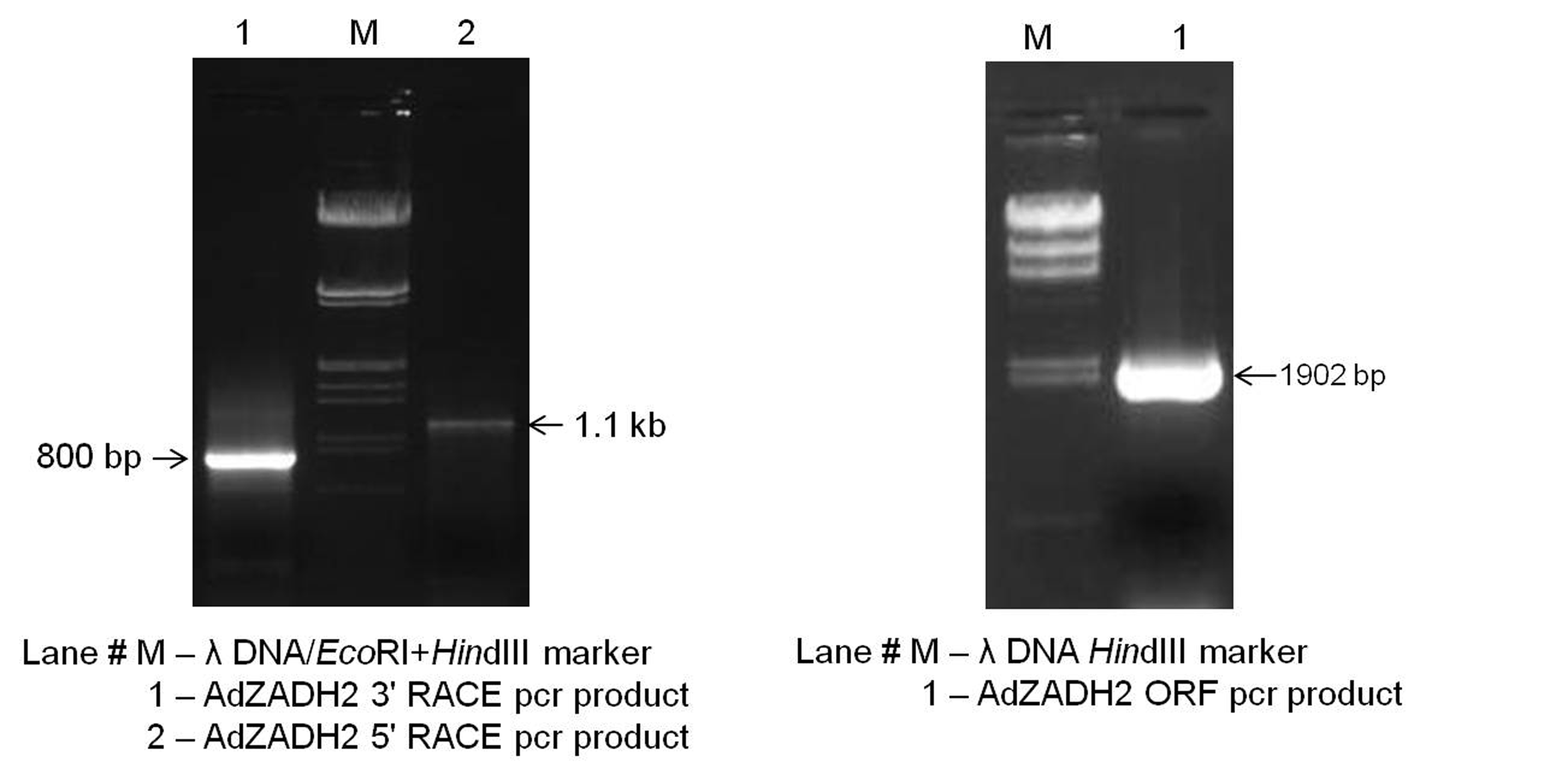


**Figure S2 (A)** Multiple sequence alignment of the deduced amino acid sequence of AdZADH2 with closely related uncharacterised Zinc-binding alcohol dehydrogenase sequences from other organisms was done by ClustalW. The genes encoding the AdZADH2 (*Arachisdiogoi*; GU785018) homologs used in the alignment were from Soybean (*Glycine max*; XP_003518845.1), tobacco (*Nicotianatomentosiformis*; XP_009612756.1), Arabidopsis (*Arabidopsis thaliana*; NP_175390.2) and Rice (*Oryzabrachyantha*; XP_006661314.1). Amino acid residues that are completely identical (100%) in all the sequences are shown by white letters on black background and gray shaded boxes indicate similar residues. Protein containing two domain, a NADB Rossmann domain and MDR domain has been represented. **(B)** Diagramatic presentation of full length AdZADH2 protein, containing N-terminal NADB Rossmann domain and C-terminal MDR domain in the coding sequence represented by closed box and flanked by 122 bp 5' UTR and 271 bp 3' UTR.


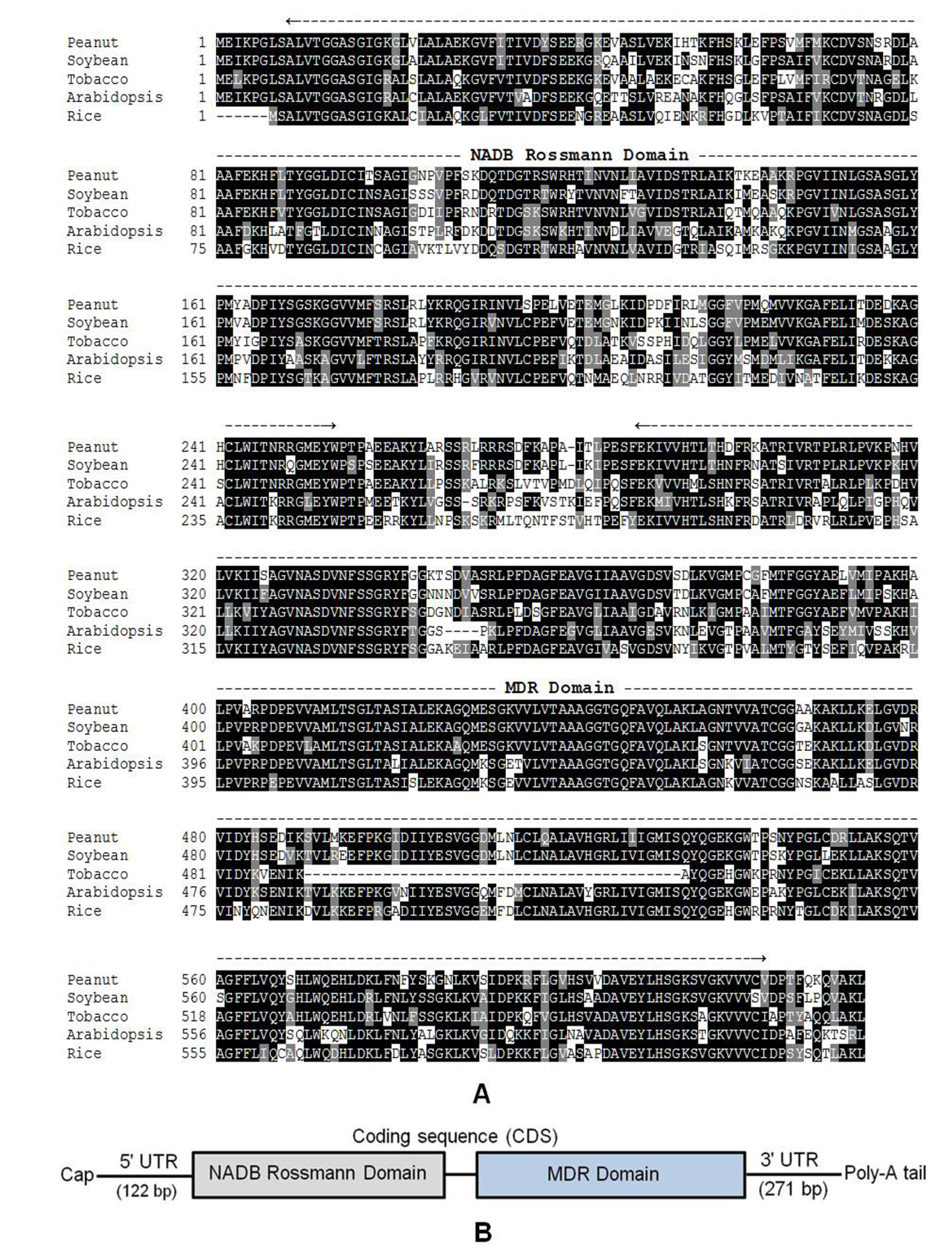


**Figure S3** Phylogenetic relationship of AdZADH2 with other zinc-binding alcohol dehydrogenase family members from different plant species constructed by neighbour-joining algorithms of MEGA 4.0 software after the multiple protein sequences alignment using the ClustalW program. Bootstrapping was performed to obtain support values for each branch.

**Figure S4**

*Brachypodiumdistachyon*gi|357158783

*Triticumurartu*gi|474089309

*Setaria italic* gi|514730798

*Oryzabrachyantha*gi|573958213

*Jatrophacurcas*gi|802563425

*Populuseuphratica*gi|743794434

*Citrus sinensis*gi|568867531

*Malusdomestica*gi|657963124

*Prunusmume*gi|645261913

*Eucalyptus grandis*gi|702392399

*Gossypium arboretum* gi|728836959

*Glycine max*gi|356500045

*Arachisdiogoi*gi|301087294

*Cicerarietinum*gi|502143066

*Medicagotruncatula*gi|657389176

*Beta vulgaris* gi|731358971

*Morusnotabilis*gi|703143208

*Brassica rapa*gi|685314573

*Camelina sativa* gi|727438327

*Arabidopsis thaliana* gi|22330139

*Vitisvinifera*gi|225428782

*Cucumismelo*gi|659112207

*Sesamumindicum*gi|747081953

*Solanumtuberosum*gi|565359231

*Nicotianatomentosiformis*gi|697117628

100

73

100

100

99

100

98

95

64

40

31

31

30

17

10

6

2

6

9

98

100

51

0.05

Multiple sequence alignment of the deduced amino acid sequence of *AdZADH2* with some previously studied zinc-binding alcohol dehydrogenase sequences from other organisms was done using default parameters of network protein sequence analysis (NPS). NADB Rossmann fold is absent in previously studied ADH genes; moreover MDR domain of *AdZADH2* shows no significant homology with other ADH proteins in the genes used in the alignment were from *Zea mays* ( ZmADH1, ZmADH2), *Oryza sativa* (OsADH1), *Solanumlycoperisicum* (SlADH1), *Arabidopsis thaliana* (AtADH1, AtADH2), *Glycine max* (GmADH1, GmADH2) and *Lotus japonicus* (LjADH1) respectively. Accession Id’s are mentioned in Table S3.

---------------------------------------------------------NADB Rossmann Domain--------------------------------------------------------
AdADH2 1 MEIKPGLSALVTGGASGIGKGLVLALAEKGVFITIVDYSEERGKEVASLVEKIHTKFHSKLEFPSVMFMKCDVSNSRDLAAAFEKHFLTYGGLDICITSA
ZmADH1 1 ....................................................................................................
OsADH1 1 ....................................................................................................
ZmADH2 1 ....................................................................................................
VvADH1 1 ....................................................................................................
SlADH2 1 ....................................................................................................
AtADH1 1 ....................................................................................................
GmADH1 1 ....................................................................................................
LjADH1 1 ....................................................................................................
GmADH2 1 ....................................................................................................
AtADH2 1 ....................................................................................................
consensus 1
---------------------------------------------------------------------------------NADB RossmannDoamin------------------------------------------------------------------
AdADH2 101 GIGNPVPFSKDQTDGTRSWRHTINVNLIAVIDSTRLAIKTKEAAKRPGVIINLGSASGLYPMYADPIYSGSKGGVVMFSRSLRLYKRQGIRINVLSPELV
ZmADH1 1 ....................................................................................................
OsADH1 1 ....................................................................................................
ZmADH2 1 ....................................................................................................
VvADH1 1 ....................................................................................................
SlADH2 1 ....................................................................................................
AtADH1 1 ....................................................................................................
GmADH1 1 ....................................................................................................
LjADH1 1 ....................................................................................................
GmADH2 1 ....................................................................................................
AtADH2 1 ....................................................................................................
consensus 101
---------------------------------------------------------------------------------MDR Domain---------------------------------------------------------------------------------
AdADH2 201 ETEMGLKIDPDFIRLMGGFVPMQMVVKGAFELITDEDKAGHCLWITNRRGMEYWPTPAEEAKYLARSSRLRRRSDFKAPAITLPESFEKIVVHTLTHDFR
ZmADH1 1 ........................MATAGKVIKCKAAVAWEAGKPLSIEEVEVAPPQAMEVRVKILFTSLCHTDVYFWEAKGQTPVFPRIFGHEA..G..
OsADH1 1 ........................MATAGKVIKCKAAVAWEAAKPLVIEEVEVAPPQAMEVRVKILFTSLCHTDVYFWEAKGQTPVFPRIFGHEA..G..
ZmADH2 1 ........................MATAGKVIKCRAAVTWEAGKPLSIEEVEVAPPQAMEVRIKILYTALCHTDVYFWEAKGQTPVFPRILGHEA..G..
VvADH1 1 .......................MSGTAGQVICCKAAVAWEAGKPLVIEEVEVAPPQAMEVRLKILYTSLCHTDVYFWEAKGQTPLFPRIFGHEA..G..
SlADH2 1 .......................MSTTVGQVIRCKAAVAWEAGKPLVMEEVDVAPPQKMEVRLKILYTSLCHTDVYFWEAKGQNPVFPRILGHEA..A..
AtADH1 1 ........................MSTTGQIIRCKAAVAWEAGKPLVIEEVEVAPPQKHEVRIKILFTSLCHTDVYFWEAKGQTPLFPRIFGHEA..G..
GmADH1 1 .............................QTIKCKAAIAWEAGKPLVIEEVEVAPPQAGEVRLKILYTSLCHTDVYFWDAK.QTPLFPRIFGHEASVG..
LjADH1 1 .......................MSTTAGQVIKCRAAVSWEAGKPLVIEEVEVAPPQAGEVRLKILYTSLCHTDVYFWEAKGQTPLFPRIFGHEA..G..
GmADH2 1 .......................MSSTAGQVIKCKA.VSWEAGKPLVIEEVEVAPPQAGEVRLKILYTSLCHTDVYFWEAKGQTPLFPRIFGHEA..G..
AtADH2 1 ........................MATQGQVITCKAAVAYEPNKPLVIEDVQVAPPQAGEVRIKILYTALCHTDAYTWSGKDPEGLFPCILGHEA..A..
consensus 201 .. ............................*... *.........*................*..*..*.. .
 ---------------------------------------------MDR Domain---------------------------------------------------------------------------------
AdADH2 301 KATRIVRTPLRLPVKPNHVLVKIISAGVNASDVNFSSGRYFGGKTSDVASRLPFDAGFEAVGIIAAVGDSVSDLKVGMPCGFMTFGGYAELVMIPAKHAL
ZmADH1 73 GIIESVGEGVTDVAPGDHVLPVFTG........ECKECAHCKSAESNMCDLLRINTDRGVMIADGKSRFSINGKPIYHFVGTSTFSEYTVMHVGCVAKIN
OsADH1 73 GIVESVGEGVTDLAPGDHVLPVFTG........ECKECAHCKSAESNMCDLLRINTDRGVMIGDGKSRFSINGKPIYHFVGTSTFSEYTVMHVGCVAKIN
ZmADH2 73 GIVESVGEGVTDVAPGDHVLPVFTG........ECKECAHCKSEESNMCDLLRINVDRGVMIGDGKSRFTISGQPIFHFVGTSTFSEYTVIHVGCLAKIN
VvADH1 74 GIVESVGEGVTDLKPGDHVLPIFTG........ECKDCAHCKSEESNLCDLLRINTDRGVMIHDGKSRFTIKGKPIYHFLGTSTFSEYTVVHVGCVAKIN
SlADH2 74 GIVESVGEGVTDLAPGDHVLPVFTG........ECKDCAHCKSEESNMCSLLRINTDRGVMLNDGKSRFSINGNPIYHFVGTSTFSEYTVVHVGCVAKIN
AtADH1 73 GIVESVGEGVTDLQPGDHVLPIFTG........ECGECRHCHSEESNMCDLLRINTERGGMIHDGESRFSINGKPIYHFLGTSTFSEYTVVHSGQVAKIN
GmADH1 69 GIVESVGEGVTHLKPGDHALPVFTG........ECGDCAHCKSEESNMCELLRINTDRGVMIHDGQSRFSKNGQPIHHFLGTSTFSEYTVVHAGCVAKIN
LjADH1 74 GIVESVGEGVTHLKPGDHALPVFTG........ECGECPHCKSEESNMCDLLRINTDRGVMISDNQSRFSIKGKPIYHFVGTSTFSEYTVLHAGCVAKIN
GmADH2 73 GIVESVGEGVTHLKPGDHALPVFTG........ECGDCPHCKSEESNMCDLLRINTDRGVMIHDSQTRFSIKGQPIYHFVGTSTFSEYTVVHAGCVAKVN
AtADH2 73 GIVESVGEGVTEVQAGDHVIPCYQA........ECRECKFCKSGKTNLCGKVRSATGVGIMMNDRKSRFSVNGKPIYHFMGTSTFSQYTVVHDVSVAKID
consensus 301 .....*....... ...*....... ............................. .................*..**..*............
 ---------------------------------------------MDR Domain---------------------------------------------------------------------------------
AdADH2 401 PVARPDPEVVAMLTSGLTASIALEKAGQMESGKVVLVTAAAGGTGQFAVQLAKLAG.NTVVATCGGAAKAKLLKELGVDRVIDY..HSEDIKSVLMKEFP
ZmADH1 165 PQAPLDK..VCVLSCGYSTGLGASINVAKPPKGSTVAVFGLGAVGLAAAEGARIAGASRIIGVDLNPSRFEEARKFGCTEFVNPKDHNKPVQEVLAEMTN
OsADH1 165 PAAPLDK..VCVLSCGISTGLGATINVAKPPKGSTVAIFGLGAVGLAAAEGARIAGASRIIGIDLNANRFEEARKFGCTEFVNPKDHDKPVQQVLAEMTN
ZmADH2 165 PEAPLDK..VCILSCGISTGLGATLNVAKPAKGSTVAIFGLGAVGLAAMEGARLAGASRIIGVDINPAKYEQAKKFGCTEFVNPKDHDKPVQEVLIELTN
VvADH1 166 PLAPLDK..VCVLSCGISTGLGATLNVAKPTKGSTVAVFGLGAVGLAAAEGARIAGASRIIGVDLNPKRYEGAKKFGVTDFVNPKDHEKSVQEVIVEMTG
SlADH2 166 PLAPLDK..VCVLSCGISTGLGASLNVAKPTKGSSVAIFGLGAVGLAAAEGARIAGASRIIGVDLNASRFEQAKKFGVTEFVNPKDYSKPVQEVIAEMTD
AtADH1 165 PDAPLDK..VCIVSCGLSTGLGATLNVAKPKKGQSVAIFGLGAVGLGAAEGARIAGASRIIGVDFNSKRFDQAKEFGVTECVNPKDHDKPIQQVIAEMTD
GmADH1 161 PAAPLDK..VCVLSCGICTGFGATVNVAKPKPGSSVAIFGLGAVAVAAAEGARVSGASRIIGVDLVSARFEEAKKFGVNEFVNPKDHDKPVQQVIAEMTN
LjADH1 166 PAAPLDK..VCILSCGICTGFGATVNVAKPKPGSSVAIFGLGAVGLAAAEGARVSGASRIIGVDLVSSRFEGAKKFGVNEFVNPKDHDKPVQEVIAEMTN
GmADH2 165 PAAPLDK..ICVLSCGICTGLGATINVAKPKPGSSVAIFGLGAVGLAAAEGARISGASRIIGVDLVSSRFEEAKKFGVNEFVNPKDHDKPVQEVIAAMTN
AtADH2 165 PTAPLDK..VCLLGCGVPTGLGAVWNTAKVEPGSNVAIFGLGTVGLAVAEGAKTAGASRIIGIDIDSKKYETAKKFGVNEFVNPKDHDKPIQEVIVDLTD
consensus 401 * *..*. ......*.............. ..........*.........*...*.......... ... ....*................*......
 ---------------------------------------------MDR Domain---------------------------------------------------------------------------------
AdADH2 498 KGIDIIYESVGG.DMLNLCLQALAVHGRLIIIGMISQYQGEKGWTPSNYPGLCDRLLAKSQTVAGFFLVQYSHLWQEHLDKLFNFYSKGNLKVSIDPKRF
ZmADH1 263 GGVDRSVECTGNINAMIQAFECVHDGWGVAVLVGVPHKDAEFKTHPMNF........LNERTLKGTFFGNYKP..RTDLPNVVELYMKKELEVEKFITHS
OsADH1 263 GGVDRSVECTGNINAMIQAFECVHDGWGVAVLVGVPHKDAEFKTHPMNF........LNERTLKGTFFGNYKP..RTDLPNVVELYMKKELEVEKFITHS
ZmADH2 263 GGVDRSVECTGNVNAMISAFECVHDGWGVAVLVGVPHKDDQFKTHPMNF........LSEKTLKGTFFGNYKP..RTDLPNVVEMYMKKELELEKFITHS
VvADH1 264 GGVDRSLECTGNVNAMISAFECVHDGWGVAVIVGVPNKDDVFKTHPINL........LNERTLKGTFFGNYKP..RSDIPAVVEKYMNKELEVEKFITHS
SlADH2 264 GGVDRSVECTGHIDAMISAFECVHDGWGVAVLVGVPHKEAVFKTHPLNF........LNERTLKGTFFGNYKP..RSDIPCVVEKYMNKELELEKFITHT
AtADH1 263 GGVDRSVECTGSVQAMIQAFECVHDGWGVAVLVGVPSKDDAFKTHPMNF........LNERTLKGTFFGNYKP..KTDIPGVVEKYMNKELELEKFITHT
GmADH1 259 GGVDRAVECTGSIQAMVSAFECVHDGWGLAVLVGVPSKDDAFKTAPINF........LNERTLKGTFYGNYKP..RTDLPSVVEKYMSGELEVDKFITHT
LjADH1 264 GGVDRAVECTGSIQAMISAFECVHDGWGVAVLVGVPNKDDAFKTHPVNF........LNERTLKGTFYGNYKP..RTDLPNVVEMYMRGELELEKFITHT
GmADH2 263 GGVDRAVECTGSIQAMISAFECVHDGWGVAVLVGVPNKDDAFKTHPVNF........LNERTLKGTFYGNYKP..RTDLPSVVEKYMNG...........
AtADH2 263 GGVDYSFECIGNVSVMRAALECCHKGWGTSVIVGVAASGQEISTRPFQL........VTGRVWKGTAFGGFKS..RTQVPWLVEKYMNKEIKVDEYITHN
consensus 501 .*.*...*..* ........................ ... ....*... .......*........ ..... ....*. ............
 ----------------------
AdADH2 597 LGVHSVVDAVEYLHSGKSVGKVVVCVDPTFQKQVAKL
ZmADH1 353 VPFAEINKAFDLMAKGEGIRCIIRMEN..........
OsADH1 353 VPFSEINTAFDLMHKGEGIRCIIRMEN..........
ZmADH2 353 VPFSEINTAFDLMLKGESLRCIMRMED..........
VvADH1 354 VPFSEINKAFEYMLKGEGLRCIIHMEP..........
SlADH2 354 LPFAEINKAFDLMLKGEGLRCIITMAD..........
AtADH1 353 VPFSEINKAFDYMLKGESIRCIITMGA..........
GmADH1 349 VPFSEINKAFDLMLKG.SIRCIIRMQE..........
LjADH1 354 VSFSEINKAFDYMLKGESIRCIIRMEE..........
GmADH2 .....................................
AtADH2 353 LTLGEINKAFDLLHEGTCLRCVLDTSK..........
consensus 601 .........................

**Figure S5**

Cloning of NADB Rossman and MDR domain in pER8 vector. Arrow indicates insert release of expected size fragment from recombinant vectors upon restriction digestion by respective enzymes.

**
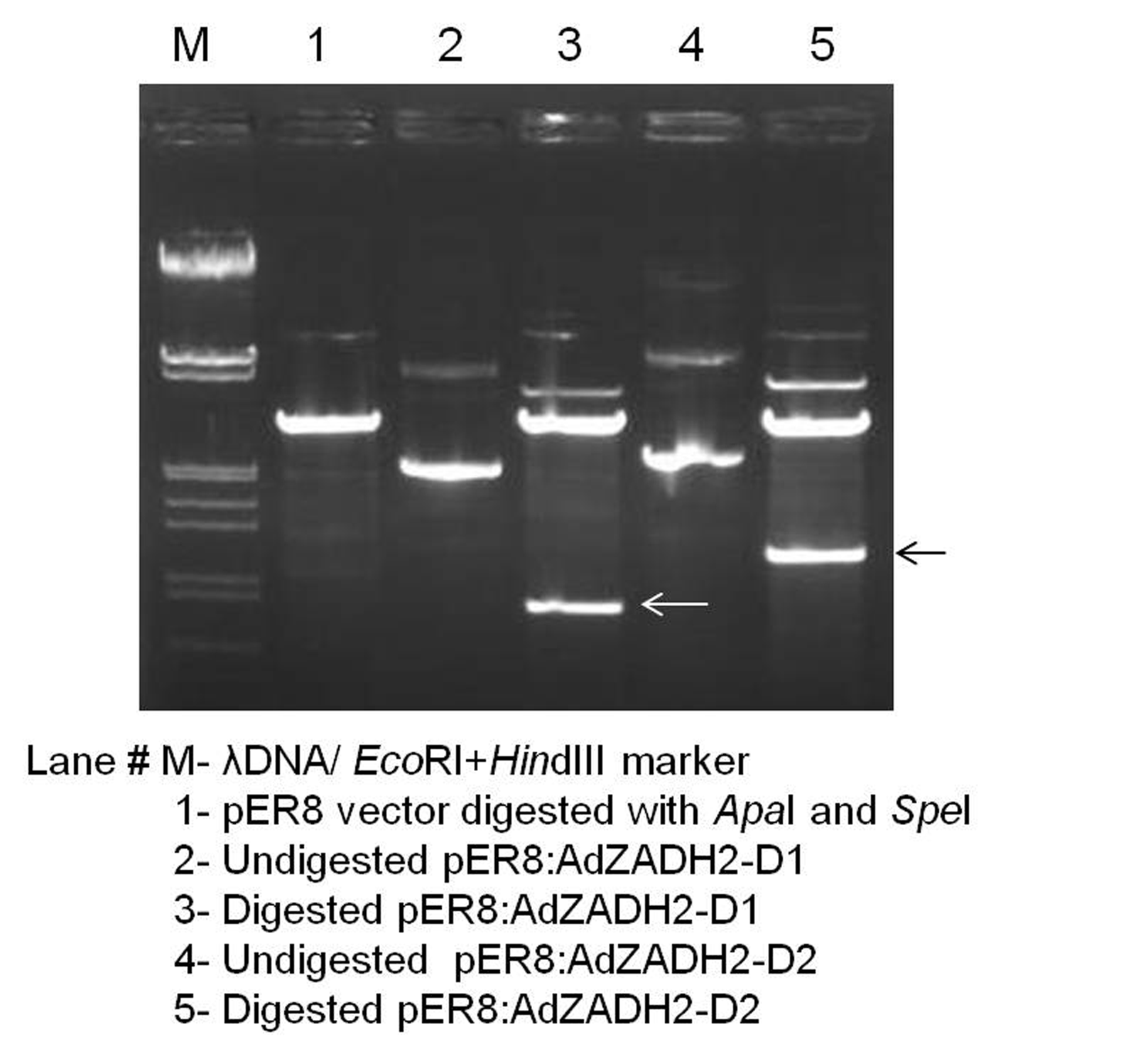
**

**Figure S6** Transient constitutive expression of*AdZADH2* induced cell death in tobacco leaf upon constitutive expression. Tobacco leaf was infiltrated with the *Agrobacterium* strain carrying the empty vector p2300 and the *AdZADH2:*p2300 and photographs was taken 96-120 hpi. HR-like cell death phenotype was observed in cDNA of AdZADH2 under 35S promoter in tobacco*.*


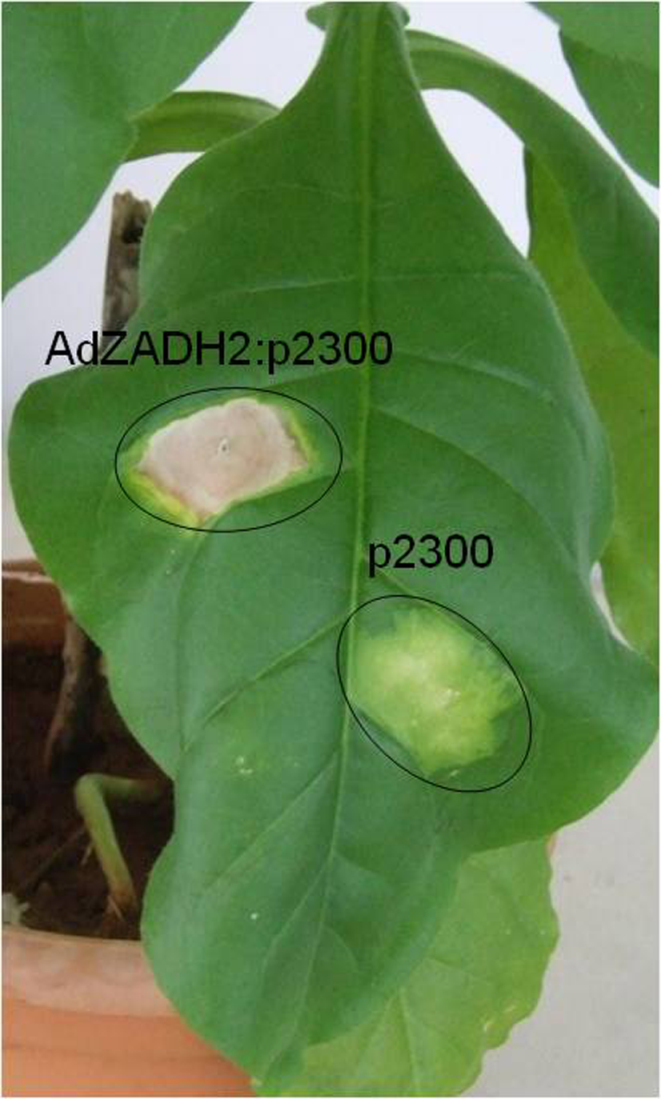


**References:**

1 Kato-Noguchi H (2001) Wounding stress inducesalcohol dehydrogenase in maize and lettuce seedlings. *Plant Growth Regul* **35**, 85–288.

2 Ismond KP (2003) Enhanced low oxygen survival in Arabidopsis through increased metabolic flux in the fermentative pathway. *Plant Physiol* **132**, 1292–1302.

3 Martinez MC, Achkor H, Persson B, Fernandez MR, Shafqat J, Farres J, Jornvall H and Pares X (1996) Arabidopsis formaldehyde dehydrogenase. Molecular properties of plant class III alcohol dehydrogenase provide further insights into the origins, structure and function of plant class P and liver class I alcohol dehydrogenases. Eur J Biochem 241, 849–857.

4 Hren M, Nikoli_c P, Rotter A, Blejec A, Terrier N, Ravnikar M, Dermastia M and Gruden K (2009) ‘Bois noir’phytoplasma induces significant reprogramming of the leaf transcriptome in the field grown grapevine. *BMC Genom* **10**, 460.

5 Tesniere C and Verries C (2000) Molecular cloning and expression of cDNAs encoding alcohol dehydrogenases from Vitis vinifera L. during berry development. *Plant Sci* **157**, 77–88.

6 Gerlach WL, Pryor AJ, Dennis ES, Ferl RJ, Sachs MM and Peacock WJ (1982) cDNA cloning and induction of the alcohol dehydrogenase gene (Adh1) of maize. *Proc Natl Acad Sci USA* **79**, 2981–2985.

7 Osterman*, JC and* Dennis*, ES (*1989*)* Molecular analysis of the Adh1-Cm allele of maize. *Plant Mol Biol* **13,**203-212.

8 Dennis ES, Sachs MM, Gerlach WL, Finnegan EJ and Peacock WJ (1985) Molecular analysis of the alcohol dehydrogenase 2 (Adh2) gene of maize. *Nucleic Acids Res* **13**, 727–743.

9 Newman KD and VanToai TT (1992) Molecular characterization of the soybean alcohol dehydrogenase gene family amplified in vitro by the polymerase chain reaction. *Plant Physiol* **100**, 489–495.

10Paul A-L and Ferl RJ (1998) Permeabilized Arabidopsis protoplasts provide new insight into the chromatin structure of plant alcohol dehydrogenase genes. *Dev Genet* **22**, 7–16.

11 Zeng T, Liu S, Luo R, Gong P, Zhao D and Fang X (2011) Cloning and expression of an alcohol dehydrogenase from Lotus japonicus and characterization of LjADH1. *Legume Genomics Genet* **2**, 6-13.

12 Uehara T, Sugiyama S, Matsuura H, Arie T and Masuta C (2010) Resistant and susceptible responses in tomato to cyst nematode are differentially regulated by salicylic acid. *Plant Cell Physiol* **51**, 1524–1536. 14

13 Xie Y and Wu R (1989) Rice alcohol dehydrogenase genes: anaerobic induction, organ specific expression and characterization of cDNA clones. *Plant Mol Biol* **13**, 53–68. 20

14 Matsumura H, Takano T, Takeda G and Uchimiya H (1998) Adh1 is transcriptionally active but its translational product is reduced in a rad mutant of rice (Oryza sativa L.), which is vulnerable to submergence stress. *Theor Appl Genet* **97**, 1197–1203. 2

15 Takahashi H, Saika H, Matsumura H, Nagamura Y, Tsutsumi N, Nishizawa NK and Nakazono M (2011) Cell division and cell elongation in the coleoptile of rice alcohol dehydrogenase 1-deficient mutant are reduced under complete submergence. *Ann Bot* **108**, 253–261. 22

16 Good AG, Pelcher LE and Crosby WL (1988) Nucleotide sequence of a complete barley alcohol dehydrogenase 1 cDNA. *Nucleic Acids Res* **16**, 7182. 23

17 Trick M, Dennis E, Edwards KR and Peacock W (1988) Molecular analysis of the alcohol dehydrogenase gene family of barley. *Plant Mol Biol* **11**, 147–160. 24

18 Hanson AD, Jacobsen JV and Zwar JA (1984) Regulated expression of three alcohol dehydrogenase genes in barley aleurone layers. *Plant Physiol* **75**, 573–581. 25

19 Gaut BS and Clegg MT (1991) Molecular evolution of alcohol dehydrogenase 1 in members of the grass family. *Proc Natl Acad Sci USA* **88**, 2060–2064. 26

20 Llewellyn DJ, Finnegan EJ, Ellis JG, Dennis ES and Peacock WJ (1987) Structure and expression of an alcohol dehydrogenase 1 gene from Pisum sativum (cv.“Greenfeast”). *J Mol Biol* **195**, 115–123. 27

21 Garvin DF, Weeden NF and Doyle JJ (1994) The reduced stability of a plant alcohol dehydrogenase is due to the substitution of serine for a highly conserved phenylalanine residue. *Plant Mol Biol* **26**, 643–655.

22 Vidal R, Lopez-Maury L, Guerrero MG and Florencio FJ (2009) Characterization of an alcohol dehydrogenase from the Cyanobacterium synechocystis sp. strain PCC 6803 that responds to environmental stress conditions via the Hik34-Rre1 two-component system. *J Bacteriol* **191**, 4383–4391.

23 Echave P, Tamarit J, Cabiscol E and Ros J (2003) Novel antioxidant role of alcohol dehydrogenase E from Escherichia coli. *J Biol Chem* **278**, 30193–30198.2

8
